# Mooney–Rivlin Parameter Determination Model as a Function of Temperature in Vulcanized Rubber Based on Molecular Dynamics Simulations

**DOI:** 10.3390/ma17133252

**Published:** 2024-07-02

**Authors:** Salvador Gomez-Jimenez, Tonatiuh Saucedo-Anaya, Carlos Guerrero-Mendez, Antonio Robles-Guerrero, Luis Silva-Acosta, David Navarro-Solis, Daniela Lopez-Betancur, Ada Rebeca Contreras Rodríguez

**Affiliations:** 1 Engineering Academic Unit, Autonomous University of Zacatecas, Avenue López Velarde 801, Zacatecas 98000, Mexico; jimenezs@uaz.edu.mx (S.G.-J.); aroblesp@uaz.edu.mx (A.R.-G.); david.navarro@uaz.edu.mx (D.N.-S.); danielalopez106@uaz.edu.mx (D.L.-B.); 2 Academic Unit of Science and Technology of Light and Matter, Autonomous University of Zacatecas, Campus Siglo XXI, Zacatecas 98160, Mexico; tsaucedo@uaz.edu.mx (T.S.-A.); guerrero_mendez@uaz.edu.mx (C.G.-M.); luis.silvaa@uaz.edu.mx (L.S.-A.)

**Keywords:** molecular dynamics, phenomenological models, advanced materials and processing, injection molding and other polymer fabrication processes, modeling and simulation

## Abstract

The automotive industry is entering a digital revolution, driven by the need to develop new products in less time that are high-quality and environmentally friendly. A proper manufacturing process influences the performance of the door grommet during its lifetime. In this work, uniaxial tensile tests based on molecular dynamics simulations have been performed on an ethylene–propylene–diene monomer (EPDM) material to investigate the effect of the crosslink density and its variation with temperature. The Mooney–Rivlin (MR) model is used to fit the results of molecular dynamics (MD) simulations in this paper and an exponential-type model is proposed to calculate the parameters $C_{1}(T)$ and $C_{2}\left(T\right)$. The experimental results, confirmed by hardness tests of the cured part according to ASTM 1415-88, show that the free volume fraction and the crosslink density have a significant effect on the stiffness of the EPDM material in a deformed state. The results of molecular dynamics superposition on the MR model agree reasonably well with the macroscopically observed mechanical behavior and tensile stress of the EPDM at the molecular level. This work allows the accurate characterization of the stress–strain behavior of rubber-like materials subjected to deformation and can provide valuable information for their widespread application in the injection molding industry.

## 1. Introduction

Elastomers are widely used in industry because of their exceptional properties. These include elasticity and durability. Synthetic rubber components are essential in automotive parts for sealing systems. Typically, elastomers are used in applications where they deform at low strain rates. Ethylene–propylene–diene monomer (EPDM) rubber is chemically stable; this type of elastomeric polymer is characterized by high elasticity and resistance to chemical media, while the fatigue strength and thus the service life of EPDM components are affected by increasing temperature and load cycles [1].

The automotive industry is entering a digital revolution, driven by the need to rapidly develop new products that integrate more efficiently and sustainable automated and autonomous technologies. In recent years, the industry involved in the production of EPDM components has implemented sustainability and carbon footprint reduction policies; to achieve these objectives, sustainable practices have been implemented for the design and manufacture of these components, using virtual prototyping tests to develop quality and environmentally friendly products [2].

The effectiveness of the solutions resulting from these numerical methods depends on the accuracy with which the mechanical properties of the rubber material can be explained, so numerous theoretical models have been developed in an attempt to describe the mechanical behavior of rubber. The macromolecular network structure of elastomers allows these materials to undergo large deformations when subjected to tensile stress, resulting in a nonlinear relationship between stress and strain. The effect of crosslinking and the temperature during the curing process is considered one of the major challenges in studying the complex mechanical behavior of amorphous crosslinked materials [3,4,5].

To better understand the influence of temperature on crosslinking and the stress–strain relationship, several physical explanations have been summarized, ranging from chain breakage in the amorphous structure to the sliding of molecules and the formation of more complex composite structures. For the complex crosslinking process during curing, it is evident that no existing model can accurately and effectively capture the dependence of the crosslink density and temperature on the characteristic tensile behavior of an elastomer in a thermodynamically consistent and numerically robust manner due to the strong nonlinearity [6].

Current models for the stress–strain (S-s) relationship consider only the idealized phenomenon, without taking into account intrinsic phenomena such as the crosslink distribution or crosslink density. Researchers in this field have proposed numerous hyperplastic constitutive models to describe the mechanical behavior of elastomers under stress; these models can be divided into two categories: The first category is based on statistical mechanics and assumes that the material consists of randomly distributed molecular chains. The second category is the phenomenological model, which applies the continuum mechanics method and assumes that the elastic potential is formed by invariants constructed by the strain or S-s ratio [7].

The properties of viscoelastic materials can be characterized as a function of the strain energy density. Models have been developed to theoretically reproduce S-s curves from experimental strain data. The mechanical behavior exhibits a high degree of nonlinearity; therefore, it is complex to establish a generalized strain energy density function that competently describes the S-s relationship experimentally.

The crosslink density is an important parameter affecting the mechanical properties of elastomeric materials, and these properties change significantly with the increasing crosslink concentration. Thermal gradients during the rubber vulcanization process strongly influence the density and distribution of crosslinks and, therefore, have a decisive influence on the mechanical properties and the final performance of the material [8]. The viscosity and elasticity of the crosslinked polymers are strongly dependent on the morphology of the structure and the length of the chain segments, as this determines the dynamics. Theoretically, adjacent chains in the amorphous structure can only move locally with the constraints imposed by the network bonds, leading to a reduction in the configurational freedom of mobility as well as an increase in local friction and intermolecular coordination.

The elasticity of rubber is mainly determined by the formation of chemical and physical crosslinks during the curing process, where the chain length and the crosslink density define the dynamics and the stored elastic entropy [9].

In phenomenological models, it is assumed that the elastic potential is formed by invariants constructed by the strain ratio or strain, and the different parameters of the elastic potential are determined based on experimental data using, for example, the Ogden model, the Yeoh model, or the Mooney–Rivlin model. In the Mooney–Rivlin model, the crosslink density is an important parameter that affects the S-s ratio; the parameters in this model are associated with intrinsic material properties such as network structures and intermolecular forces [10,11,12].

Several investigations have been reported using a molecular dynamics simulation on polymer properties, which explore the kinetic properties of the EPDM considering the free volume and crosslink density. For example, Lengger et al. [11] use the Mooney–Rivlin model with photoelasticity for experimental measurements to determine the evolution of cure-dependent material parameters. Vishvanatperumal et al. [13] determine the crosslinking density by studying the experimental stress–strain behavior with finite element analysis by fitting the experimental data to the Mooney–Rivlin model. Morovati et al. [14] studied the effect of fatigue stress on elastomers, providing information on the complexity of the constitutive behavior of the crosslinked polymers. Liu et al. [12] proposed a hyperplastic constitutive model that shows good agreement with the experimental data obtained in elastomeric materials subjected to large deformations. Using molecular dynamics simulations, Wang Y. et al. [5] found that the crosslinking produced in the side chains influences the stiffness and enhances the inhibitory effect on the diffusion properties; the results of the study allow for an advance in the understanding of the microscopic aspects underlying the performance of EPDMs. Wang Ao et al. [15] used time–temperature superposition and varying strain rates in molecular dynamics simulations and the data obtained from the simulations were used to predict the constants of Rouse’s exponential phenomenological model to determine the rheological properties of EPDMs.

It is important to note that the methodology of constructing crosslinked models using MD simulations has undergone rapid development in recent years. Initially, Yarovsky and Evans [16] proposed a static approach in 2002, in which the crosslinked bonds were created at a time forming a low-molecular-weight crosslinked network based on a predefined fixed radius. In this context and years later, Wu and Xu [17] created a small-scale crosslinked model using a dynamic method of iteratively creating new bonds with a fixed shear reaction radius by performing energy minimization and molecular dynamics (MM/MD) after each reaction. On the other hand, Varshey et al. [18] simulated the crosslinking reaction by proposing a stepwise method by constantly increasing the shear reaction radius, followed by MM/MD simulations, forming a relatively large crosslinked model. In general, to simulate the crosslinking process of EPDM, crosslinking should be added periodically by implementing some stepwise or multi-step algorithm as this is key to simulate the crosslinking process of EPDMs; the most prominent works in this regard to mention a few have been Papanikolaou et al., Wang et al. and van Duin et al. [2,19,20].

In this research, the study of the S-s relationship using the Mooney–Rivlin model considering the crosslink density in the amorphous structure of EPDMs through molecular dynamics modeling is approached. The methodology proposed in this work considers the molecular dynamics of the polymer chains in the amorphous structure on the macroscopic behavior for the mechanical characterization of EPDM subjected to large deformations and to provide a phenomenological model for their design in applications for the automotive industry.

## 2. Models and Methods

### 2.1. Molecular Dynamics Model

**Figure 1:**
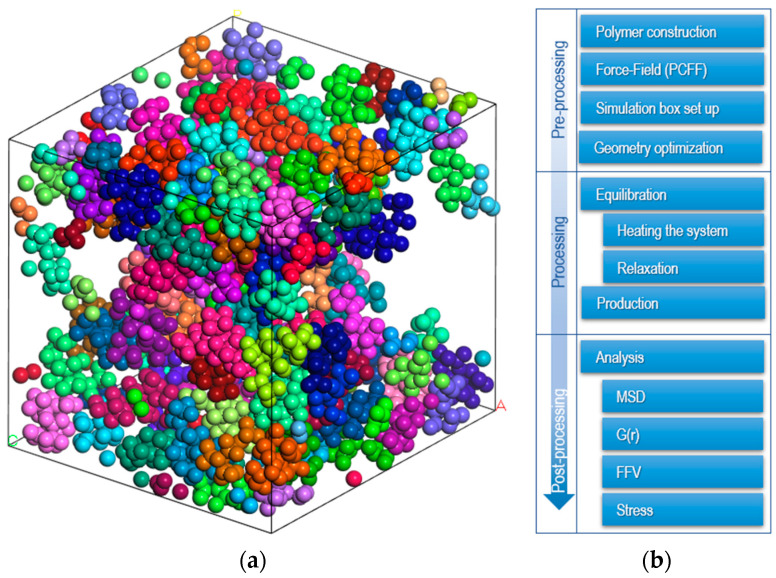
(**a**) Random distribution of EPDM polymer chains in a simulation box (each color represent different backbone). (**b**) Procedure for molecular dynamics simulation.

Molecular dynamics (MD) simulations assume a non-overlapping configuration of all amorphous EPDM polymer chains, with five chains consisting of 1000 monomers randomly distributed in a simulation box (Figure 1a). Three-dimensional periodic boundary conditions are applied to eliminate surface effects.

The force field implemented in this work was calculated by condensed-phase optimized molecular potentials for atomistic simulation studies COMPASS. A simplified model of the Coulomb and van der Waals force interactions is used for the COMPASS force field [21,22]. The van der Waals interactions hold atoms of opposite charge at a certain distance from each other. The summation methods for electrostatic (Coulombic) and van der Waals interactions were calculated with the Atom-based and Ewald algorithms, respectively [19,21].

The interaction potential function in a system between polymer chains and its parameters associated with the valence terms represent the internal bond coordinates (b), angle (θ), torsion angle (φ), and out-of-plane angle (χ). The vibrational frequencies and the structural variations associated with the changes in their conformation are related to each other in terms of cross-coupling. These terms include combinations of two or three internal coordinates considering the bond angle potential ($E_{ba}$), the in-plane dihedral rotation potential ($E_{dh}$), the out-of-plane vibrational potential ($E_{inv}$), and the cross term ($E_{cross}$). Non-bonding interactions are described by Lennard-Jones 9-6 potential for the van der Walls term ($E_{vdW}$) and a Coulomb function for an electrostatic interaction ($E_{coul}$). The functional terms of COMPASS are given by Equation (1):

(1) $$E_{total}=E_{vdW}+E_{coul}+E_{ba}+E_{dh}+E_{inv}+E_{cross}$$

where $E_{total}$ is the total energy of the system and $E_{vdW}$ is the potential energy due to intermolecular forces calculated by Equation (2):

(2) $$E_{vdW}=\sum_{ij}\varepsilon_{ij}\left[{2\left(\frac{\sigma_{ij}}{r_{ij}}\right)}^{9}-{3\left(\frac{\sigma_{ij}}{r_{ij}}\right)}^{6}\right]\;$$

where the subscripts ij in the summations represent the potential energy interactions, $\varepsilon_{ij}$ represents the potential energy parameter, $\sigma_{ij}$ represents the finite distance when the potential energy between the molecules becomes zero, $r_{ij}$ denotes the cut-off distance between atoms i and j and $E_{coul}$ is the Coulomb electrostatic potential that can be expressed by Equation (3):

(3) $$E_{coul}=\sum_{ij}\frac{q_{i}q_{j}}{r_{ij}}\;$$

where $q_{i}$ and $q_{j}$ are the fixed partial charges between atom i and j within the same molecule. $E_{ba}$ is the bond angle potential shown in Equation (4):

(4) $$E_{ba}=\sum_{\theta }\left[k_{2}{\left(\theta -\theta_{0}\right)}^{2}+k_{3}{\left(\theta -\theta_{0}\right)}^{3}+k_{4}{\left(\theta -\theta_{0}\right)}^{4}\right]\;$$

where θ represents the energy associated with the angle, the value $\theta_{0}$ represents the equilibrium, and k, $k_{1}$, $k_{2}$, and $k_{3}$ are force constants. $E_{dh}$ is the dihedral rotation potential within the molecule defined by Equation (5):

(5) $$E_{dh}=\sum_{\phi }\left[k_{1}\left(1-\cos\phi \right)+k_{2}\left(1-\cos2\phi \right)+k_{3}\left(1-\cos3\phi \right)\right]\;$$

where ϕ represents the torsional angle. The out-of-plane vibrational potential $E_{inv}$ is represented by Equation (6)

(6) $$E_{inv}=\sum_{\chi }k_{x}\chi^{2}\;$$

Wilson out-of-plane χ internal coordinates are shown in Equation (7) $E_{cross}$, which includes the cross-coupling terms to calculate the vibrational frequencies and structural variations associated with conformational changes.

(7) $$\begin{array}{c} E_{cross}=\sum_{b,b\prime }k\left(b-b_{0}\right)\left(b\prime -{b\prime }_{0}\right)+\sum_{b,\theta }k\left(b-b_{0}\right)\left(\theta -\theta_{0}\right)\\ +\sum_{b,\phi }k\left(b-b_{0}\right)\left(k_{1}cos\phi +k_{2}\cos2\phi +k_{3}\cos3\phi \right)\\ \begin{array}{c} +\sum_{\theta ,\phi }k\left(\theta -\theta_{0}\right)\left(k_{1}cos\phi +k_{2}\cos2\phi +k_{3}\cos3\phi \right)\\ +\sum_{\theta ,\theta \prime }k\left(\theta -\theta_{0}\right)\left(\theta \prime -{\theta \prime }_{0}\right)+\sum_{\theta ,\theta \prime ,\phi }k\left(\theta -\theta_{0}\right)\left(\theta \prime -{\theta \prime }_{0}\right)cos\phi \end{array} \end{array}$$

The velocity-Verlet algorithm is employed to integrate the equations of motion, with a time step integration set to 1 fs, and the initial velocities were chosen randomly from the Boltzmann distribution. An Andersen-type thermostat and barostat were used to control the temperature and pressure.

There is no general methodology for equilibrating the MD systems, but there are guidelines to follow (see Figure 1b).

In this study, using the methodology proposed by Gómez et al. [4], the system was equilibrated in two stages with four frames (replicas), optimizing the topology for each frame (pre-equilibration).

The generated frames are equilibrated through various conditions to bring them to a constant energetic state, defining conditions related to variables such as energy, pressure, volume, and temperature.

In the first equilibrium stage, simulated annealing with a constant energy and volume NVE ensemble is carried out in a temperature range of 350 to 500 K with 100 cycles and 10 ramps per cycle, a time step 1 fs, with 200,000 steps.

In the second step, the system was relaxed at a constant volume and controlled temperature NVT ensemble using the Andersen thermostat. On the obtained trajectories, to equilibrate the pressure variation, NPT ensemble simulations (100 MPa) were performed to equilibrate the simulation box size and to obtain the correct system density for production, both simulations with 20 ns with a time step of 1 fs, and the process was repeated in the temperature range from 428 K to 478 K with 10 K steps.

The method described below is based on earlier work by Papanikolaou et al. and adopted by Wang Y et al. [2,5].The simulation was based on the calculation of all crosslinks and the distances between the reactive atoms within a preset cut-off range between 0.4 nm and 1 nm and a crosslinking limit of 90%. The crosslinking kinetics were performed randomly and periodically and covalent terms were created for crosslinking. The new bonds were activated by alternating equilibrium distances and force coefficients to avoid missing atoms in the simulation box. The lower bound cut-off distance was increased by 0.025 nm at each iteration, and the distance between the reactive atoms was rechecked. The simulation box was equilibrated using three-dimensional periodic boundary conditions to eliminate surface effects on dynamic crosslinking. The procedure continues until the reactive pairs are exhausted within the pre-assigned cut-off distance, and the crosslinking limit is reached.

**Figure 2:**
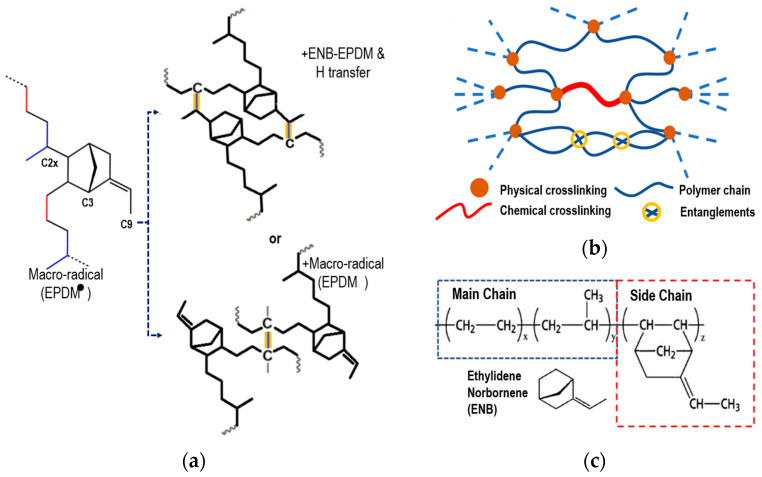
(**a**) Scheme of physical crosslinking rubber with peroxide of ENB-EPDM (+ENB-EPDM and H transfer or + macro-radical (EPDM)). (**b**) Schematic representation of the molecular structure of a cured elastomer. (**c**) Chemical structure of ethylene–propylene–diene monomer.

Next, the chemical crosslinking process of EPDM is briefly explained, based on the research of Wang et al. [5,19], to model the process with a third 5-ethylidene-2-norbornene (ENB) monomer. van Duin et al. [20] and Papanikolaou et al. [2] showed that peroxide free radicals mainly capture the tertiary hydrogen (C2x) of the main chain and the hydrogens at the C3 and C9 positions of ENB, similar to Papanikolaou et al. The positions are shown schematically in Figure 2a. In EPDM, the main chain-to-side-chain ratio is approximately 9:1. Zachary et al. [23] showed that the reaction rate of C3 in the monomer was 90%, while that of C9 was only 10%. The C2x atom of the main chain and the C3 atom of the side chain will be where the crosslinking occurs. The chemical crosslinking with the C9 atom was omitted because its reaction rate is quite low compared to the previous ones.

Physical crosslinking occurs through weak interactions. These interactions are due to electrostatic and van der Waals forces that influence the gelation and elasticity of the polymer. Physical crosslinking in elastomers largely determines the mechanical properties of the elastomer and provides stability to the material. Figure 2b schematically shows chemical crosslinking and physical crosslinking.

After the initial models are sufficiently equilibrated to simulate the EPDM curing process, as described in the previous paragraph, curing is added periodically by implementing a stepwise algorithm or multi-step subroutine as described below: Step 1: all distances between reactive atoms were calculated and crosslinks were created between pairs that were within a predetermined cutoff distance. Step 2: continuously, the covalent terms of the formed crosslinks were created and the hydrogen atoms were removed from the newly formed pairs. Step 3: the new bonds were turned on in a stepwise manner by alternating the bond equilibrium distances and force coefficients to avoid atoms missing from the simulation box due to large atomic forces. Step 4: next, an NPT simulation was performed and the simulation box was further equilibrated before the distances between the reactive atoms were checked again. Step 5: the procedure was repeated until there were no other reactive pairs within the pre-assigned cutoff distance.

Subsequently the EPDM simulation box was deformed. Uniaxial stress simulations in the unit cell are performed by increasing the length along the loading direction (strain) at each MD step. A value of 1 × 10^8^ s^−1^ was assigned for the strain rate, which is a typical value in MD simulations due to the small-time scale set and is impossible to implement in a real physical system according to experimental standards. The volume of the simulation box during the deformation process is constant, the EPDM is considered incompressible, with a Poisson’s ratio equal to 0.5, and the pressure in the transverse directions is considered constant, using a barostat to regulate it. The strain limit is determined when it reaches a value of 200%. The stress–strain curves were obtained for the different temperature sets (ranging from 428 K to 478 K with 10 K steps).

Viral expression is a widely used method in atomistic calculations for the intramolecular stress tensor [24,25,26], given in Equation (8), and this method provides a measure of the mechanical interactions between molecules in a continuous form:

(8) $$\sigma_{i}=-\frac{1}{V}\left[\left(\sum_{i=1}^{N}m_{i}\left(v_{i}v_{i}^{T}\right)\right)+\left(\sum_{i=1}^{N}r_{ij}f_{ij}^{T}\right)\right]\;$$

where index i runs over all particles 1 through N; $m_{i}$, $v_{i}$, and $f_{ij}$ denote the mass, velocity and force acting on particle i; and V denotes the volume of the system. To calculate the i−th contribution of forces $f_{i}$ acting on the system from position $r_{i}$, the notation $r_{ij}$ is used, which indicates the position and the particular force that contributes to the total sum of forces $f_{ij}$ on i.

### 2.2. Phenomenological Models

The literature cites several mathematical models to describe elasticity in rubbery materials from a phenomenological approach. These models have constants that are estimated from experimental data by nonlinear regression. The stress–strain curves can be fitted using the semi-empirical formulation of the Mooney–Rivlin model.

The Mooney–Rivlin model can have 2, 3, 5, and 9 parameters to describe the behavior of rubber-like materials [27,28]. The potential strain energy function, W, of an isotropic material, is usually formulated in terms of three invariants of the strain relations. The principal invariants, $I_{1}$, $I_{2}$, and $I_{3}$, of the right Cauchy–Green deformation tensor are considered. The deformation energy potential functions are assumed to have polynomial or reduced polynomial forms. The polynomial model for an incompressible elastomer can be expressed in general terms as follows in Equation (9) [29]:

(9) $$W=\sum_{i+j}^{N}C_{ij}{\left(I_{1}-3\right)}^{i}{\left(I_{2}-3\right)}^{j}\;$$

where W is the work of deformation per unit volume or the elastically stored free energy density, $C_{ij}$ are the empirically determined material constants and $I_{1}$ and $I_{2}$ are the first and second Green’s deformation invariants, respectively.

For incompressible rubber materials, the expansion of the Mooney–Rivlin model for two parameters can be expressed as follows [30].

(10) $$W=C_{1}\left(I_{1}-3\right)+C_{2}\left(I_{2}-3\right)\;$$

Green’s deformation invariants are related to strain ratios (SR) in the three main directions by:

$$I_{1}=\lambda_{1}^{2}+\lambda_{2}^{2}+\lambda_{3}^{2}$$

$$I_{2}=\lambda_{1}^{2}\lambda_{2}^{2}+\lambda_{2}^{2}\lambda_{3}^{2}+\lambda_{3}^{2}\lambda_{1}^{2}$$

For incompressible materials, it is considered that

$$I_{3}=\lambda_{1}^{2}\lambda_{2}^{2}\lambda_{3}^{2}$$

where $\lambda_{1}$, $\lambda_{2}$ and $\lambda_{3}$ are the strain ratios in the three principal directions, considering the incompressibility ratio for uniaxial stress [31]:

$$\lambda_{1}\lambda_{2}\lambda_{3}=1$$

$$\lambda_{2}^{2}=\lambda_{3}^{2}=\lambda^{-1}\;\mathrm{and}\;\sigma_{2}=\sigma_{3}=0$$

$$\lambda_{1}=\lambda$$

The crosslinking and entanglement effects of elastomeric polymers are represented by the model parameters $C_{1}$ and $C_{1}$, respectively.

In rubbery polymers, elasticity is determined by the crosslink density resulting from the vulcanization process of the elastomer. For isotropic incompressible elastomers, the elastic stored free energy density, induced by the strain–energy relationship, is given by the specific phenomenological model of the Mooney–Rivlin Equation (11).

(11) $$W=C_{1}\left(\lambda_{1}^{2}+\lambda_{2}^{2}+\lambda_{3}^{2}-3\right)+C_{2}\left(\lambda_{1}^{-2}+\lambda_{2}^{-2}+\lambda_{3}^{-2}\right)\;$$

Given these relationships, Equation (11) becomes:

(12) $$W=C_{1}\left(\lambda^{2}+2\lambda^{-1}-3\right)+C_{2}\left(\lambda^{-2}+2\lambda -3\right)\;$$

The Kirchhoff stress tensor and the Green’s strain tensor are used to determine the general state of the tension with the following equation:

(13) $$t_{ij}=\frac{\partial W}{\partial I_{1}}\frac{\partial I_{1}}{\partial \gamma_{ij}}+\frac{\partial W}{\partial I_{2}}\frac{\partial I_{2}}{\partial \gamma_{ij}}+\frac{\partial W}{\partial I_{3}}\frac{\partial I_{3}}{\partial \gamma_{ij}}\;$$

where $t_{ij}$ is the Kirchhoff stress tensor and $\gamma_{ij}$ is Green strain tensor.

For an incompressible material subjected to uniaxial tension, the stress σ is determined by deriving Equation (12) in terms of the strain ratio:

(14) $$\frac{dW}{d\lambda }=2\left(\lambda -\lambda^{-2}\right)\left(C_{1}+\frac{C_{2}}{\lambda }\right)$$

Taking dW/dλ=σ into account and rearranging it, we finally have:

(15) $$\sigma =2\left(\lambda -\frac{1}{\lambda^{2}}\right)\left(C_{1}+\frac{C_{2}}{\lambda }\right)$$

To determine the parameter $C_{1}$, Equation (16) is used. This equation includes the crosslink density $N_{c}$ of the vulcanized EPDM [7,13].

(16) $$C_{1}=\frac{RT}{2}N_{c}$$

In the physics of entangled networks, there is currently no sufficiently rigorous and constructive formalism that adequately describes entanglement for networks of macroscopic size. Several researchers have proposed that $C_{2}$ takes on a value of zero because there is no formal equation for determining the parameter $C_{2}$ [32].

Given the above considerations, the Mooney–Rivlin model can accurately describe the mechanical behaviour of EPDM in components subjected to less than 200% deformation.

## 3. Results and Discussion

### 3.1. Molecular Dynamics Simulation

**Figure 3:**
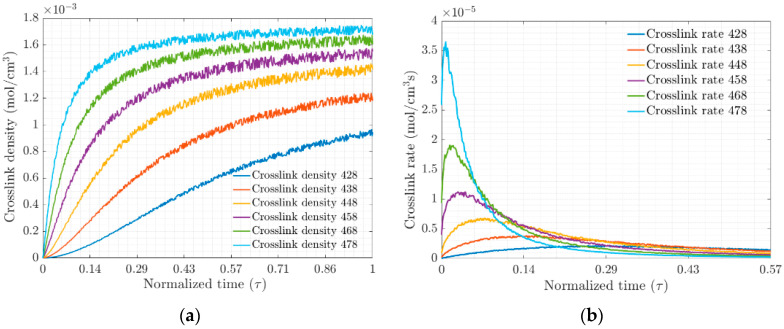
(**a**) Crosslink densities and (**b**) Rate of crosslink density versus normalized time, respectively.

The crosslink density $\left(N_{c}\right)$ and crosslink rate $\left({\dot{N}}_{c}\right)$ for each of the test temperatures, Figure 3a and Figure 3b, respectively, were obtained from the MD simulation of the EPDM composite. These results are plotted on a normalized time scale $\left(\tau \right)$; they show that the density and rate are strongly temperature-dependent. The ratio of the number of crosslinks formed to the probability of the total possible bond formation defines the degree of vulcanization and will be a function of the active positions in the system, electrostatic interactions, van Ver Waals forces and entanglement chains.

**Figure 4:**
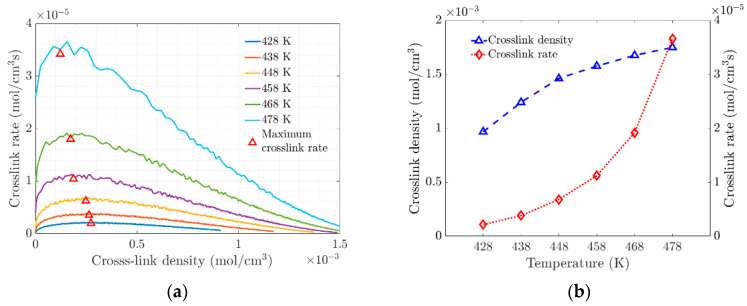
(**a**) Rate of crosslink density versus Crosslink density. (**b**) Variation of Crosslink Density and Crosslink Density Rate with Temperature.

The change in the slope of the crosslinking rate curves marks the gel point at the transition of the amorphous material (see Figure 4a). This transition at the gel point is due to increased crosslinking, which limits molecular movement through the polymer network. At the molecular level, the vulcanization process is characterized by chain polymerization and crosslinking, which cause significant changes in the mechanical properties, as shown macroscopically. Figure 4b shows the evolution of the density and rate of crosslink formation as a function of temperature. At higher temperatures, the polymer network becomes saturated with crosslinks, which affects the elasticity of the elastomer.

**Figure 5:**
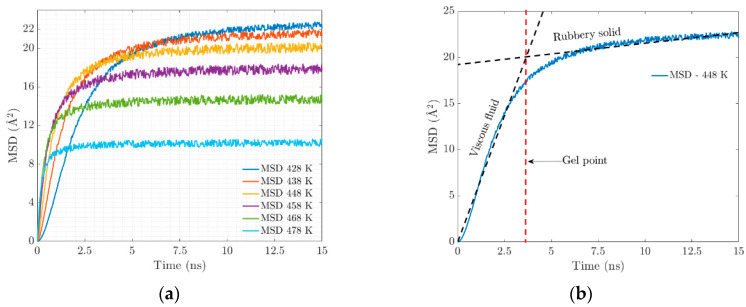
(**a**) Mean square displacement. (**b**) Mechanical behavior over simulation time.

At the molecular level, the vulcanization process is characterized by the formation of chains and crosslinking, which causes substantial changes in the mechanical properties that are observed macroscopically. At higher temperatures, the polymeric network becomes saturated with crosslinks, which will have an impact on the resulting properties of the elastomer. An important parameter is the mean squared displacement (MSD). Figure 5a shows a clear trend of the MSD of EPDM decreasing with the increasing temperature. This can be attributed to the combined effect of two key factors: the restrictions on chain movement due to crosslinking and the strong interactions at the bond interface.

**Figure 6:**
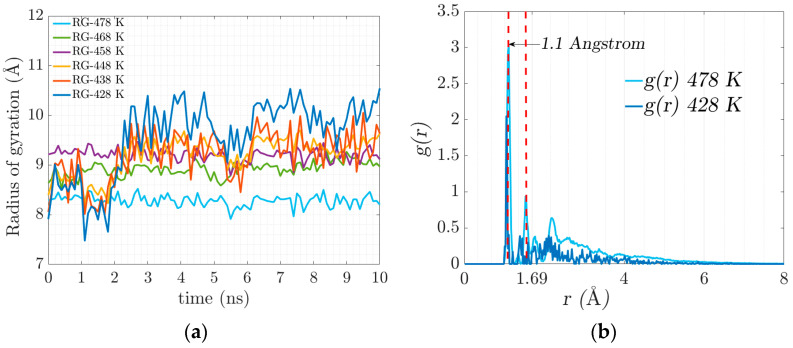
(**a**) Radius of gyration evolution for the trajectories of the different temperatures. (**b**) Radial distribution function, (or pair correlation function) g(r).

As crosslinking increases and electrostatic interactions occur, the chains begin to intertwine, resulting in the stiffening of the geometrically constrained network. The movement (diffusion) of the polymer chains is restricted, and the confinement leads to the coexistence of an amorphous crosslinked structure forming a gel state. Figure 6a illustrates the evolution of the radius of gyration in crosslinking, which is associated with the stiffness of the resulting system that manifests itself with the progressive increase in elasticity.

At the temperature of 428 K, there is greater flexibility in the main chain, which is expected to favor the formation of crosslinks. At 478 K, an agglomeration of side chains is formed. There is no significant conformational change in the main chain due to the low availability of free volume.

The radial distribution function denoted by g(r) defines the probability of finding a certain distance between two atoms of different polymeric chains where there is a physical connection by electrostatic forces. Figure 6b represents the radial distribution function where the electrostatic equilibrium distance (physical bonds) is 1.1 Angstrom, represented in the figure with the dotted red line. The value of the radial function increases with temperature.

**Figure 7:**
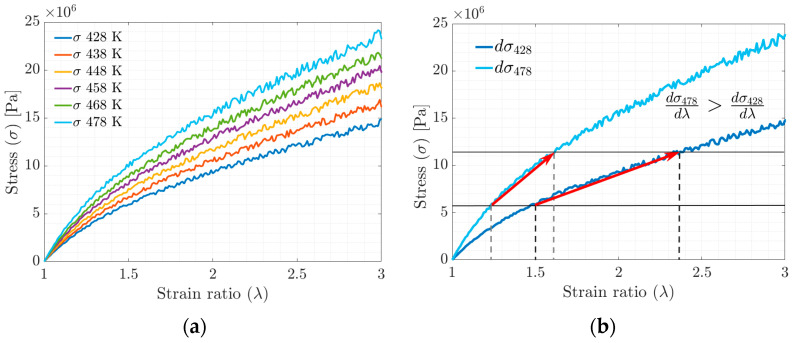
(**a**) Stress–strain ratio behavior evolution curve. (**b**) Stress–strain ratio curve between maximum and minimum simulation temperatures.

In the stress–strain ratio curves obtained by stretching in the MD simulation (Figure 7a,b), an increase in stiffness is observed concerning the increase in the tested temperature. The increase in stiffness is related to the ability of the polymer chains to slide with each other (the higher the crosslinking density, the lower the mobility) and the rearrangement of the polymer chains in the available free volume fraction.

The free volume is the space not occupied by the polymer chains within the unit cell and decreases with the increasing crosslink density and chain entanglement.

The lower the free volume fraction (FFV), the more difficult it is for the chains to rearrange, limiting their mobility, which can be seen macroscopically as an increase in stiffness.

Understanding stress behavior in terms of free volume implies that for conformational changes to occur in the backbone, there must be space available to which the molecular segment can move, implying a deformation that can be observed macroscopically.

Due to the conformational rearrangement induced by uniaxial deformation, when the minimum value of the FFV is reached, the chains can no longer slide, thus restricting the molecular motion, which is observed as an increase in the slope of the S-s plot.

The concept of free volume explains the dependence of the mobility or conformational rearrangement of molecular chains on temperature and plays an important role in the mechanics of polymers.

The FFV can be calculated using Equation (17) where $V_{f}$ is the free volume and $V_{o}$ is the volume occupied by the molecular chains in the unit cell.

(17) $$FFV=\frac{V_{f}}{V_{f}+V_{o}}$$

**Figure 8:**
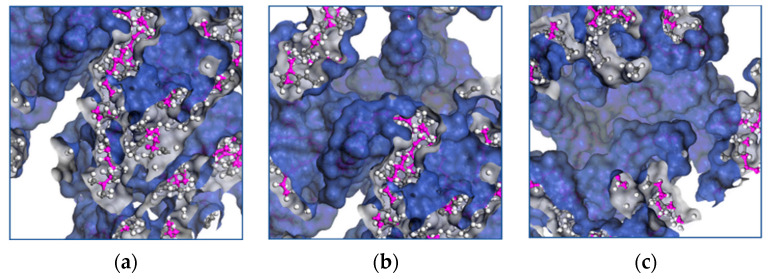

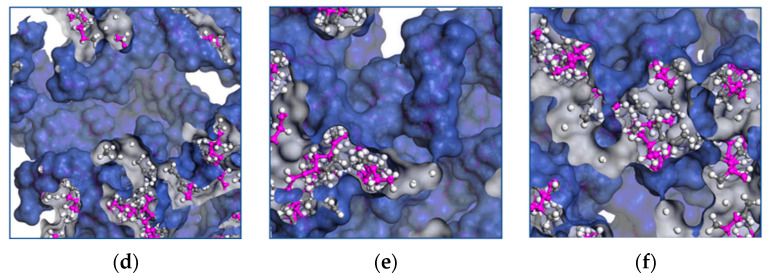
Distribution of volume occupied by EPDM polymer chains at temperatures of (**a**) 428 K, (**b**) 438 K, (**c**) 448 K, (**d**) 458 K, (**e**) 468 K and (**f**) 438 K.

Molecular transport occurs when a chain moves into voids larger than the critical volume. The cooperative motion of neighboring atoms creates the voids by redistributing the free volume. The free volume provides further insight into the mechanical properties of EPDM, and Figure 8a–f shows the influence of the temperature on the distribution of the occupied volume as it increases.

Table 1 shows the calculated values of the free volume fraction (Equation (17)) for the different simulation temperatures.

### 3.2. Phenomenological Model

Given the results obtained from the MD simulations, the Mooney–Rivlin (MR) model, Equation (15), was employed to determine the material constants.

Equation (18) minimizes the total squared error between the data obtained from the MD simulation and the data fitted by the MR model, which give N pairs of data $\left\{\lambda_{i},\;\sigma_{i}\right\},i=1,\;\ldots ,\;N$, to determine $C_{1}$ and $C_{2}$.

(18) $$e=\sum_{i=1}^{N}{\left\{\sigma_{MR}\left(\lambda_{i}\right)-\sigma_{iMD}\right\}}^{2}$$

**Figure 9:**
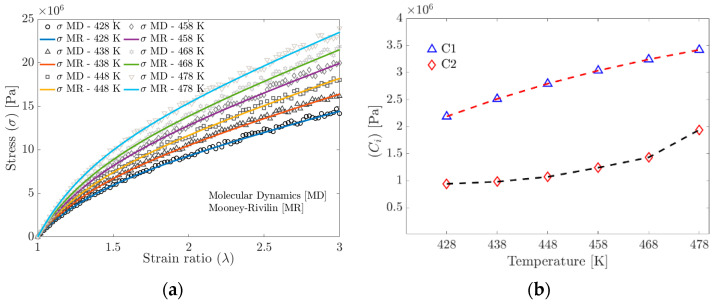
(**a**) Tensile strength curves obtained by Molecular Dynamics and curves fitted to the Mooney–Rivlin model. (**b**) Evolution of the parameters of the Mooney–Rivlin model as a function of temperature.

Figure 9a shows the stress obtained from the MD simulation and the stress calculated using the material parameters $C_{1}$ and $C_{2}$ fitted with the MR model. Figure 9b shows the $C_{1}$ and $C_{2}$ trends from the Mooney–Rivlin model fitted to the MD simulation data. It is widely documented that the $C_{1}$ parameter depends on the crosslinking density.

To calculate the temperature-dependent crosslink density, $N_{c}\left(T\right)$, the MD simulation results (Table 1) are fitted to an order-two polynomial model, Equation (19). The proposed model is limited to the range (350 K–480 K), where the curing rate is greater than zero and the temperature is below the point where physical curing (due to electrostatic interactions) begins to degrade.

(19) $$N_{c}\left(T\right)=a_{0}+a_{1}T+a_{2}T^{2}$$

where $N_{c}\left(T\right)$ is the temperature-dependent crosslink density, $a_{0}$, $a_{1}$ and $a_{2}$ are the parameters of the tested material and T is the temperature within the range to evaluate the crosslink density.

In this research, a modified empirical model, Equation (19), was proposed to calculate the $C_{1}(T)$ Equation (20) parameter of the Mooney–Rivlin model, which takes into account the temperature-dependent variation of the crosslinking density $N_{c}\left(T\right)$.

(20) $$C_{1}\left(T\right)=\frac{RT}{2}N_{c}(T)$$

where the units of $C_{1}$ are Pa, R in $MPa\cdot {cm}^{3}/mol\cdot K$, crosslink density $N_{c}\left(T\right)$ in $mol/{cm}^{3}$ and the absolute temperature (T) in Kelvin.

**Figure 10:**
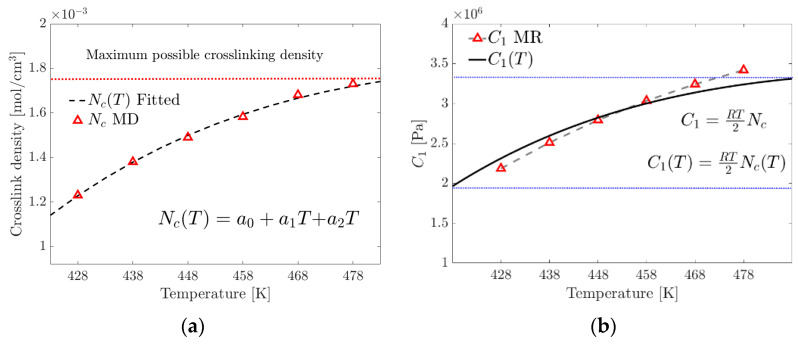
(**a**) Fitting of the proposed crosslink density model. (**b**) Contrasting the $C_{1}$ parameter of the MR model with that calculated using the proposed exponential model (the blue dotted lines represent the limits of the studied range).

Figure 10a shows a maximum limit of the possible crosslink formation density and, therefore, the existence of the maximum tensile stress before the reversibility or breakage of the physical crosslinks occurs due to the increase in temperature. Figure 10b graphically compares $C_{1}$ of the MR model and $C_{1}(T)$ of the proposed model.

Several investigations report that the $C_{2}$ parameter is related to entanglements, weak interactions and the volume fraction occupied by the polymer networks.

The mechanical strength of polymers is significantly affected by the conformational reorganization of the molecular chains as a function of temperature.

An empirical model proposed in this research to determine the $C_{2}$ parameter in Equation (21) includes the volume fraction occupied by the increase in the formation of physical crosslinks. At the macroscopic level, a change in the gradient of the stress–strain ratio occurs.

(21) $$V_{O}(T)=Ae^{\left(\frac{-E}{RT}\right)}$$

$V_{O}\left(T\right)$ is the fraction of the occupied volume as a function of temperature described by an Arrhenius-type model, where A is the pre-exponential factor and E is the specific activation energy, and this term comes from the phenomenological model of curing (Isayev–Deng) obtained by fitting the conversion fraction curve [33,34,35], a term widely used in the study of rubber vulcanization.

(22) $$C_{2}(V_{O})=S_{0}+T_{i}e^{\left[R{\cdot V}_{O}(T)\right]}$$

where $V_{O}\left(T\right)$ is the occupied volume fraction as a function of temperature and $S_{0}$ is a fitting parameter in MPa.

**Figure 11:**
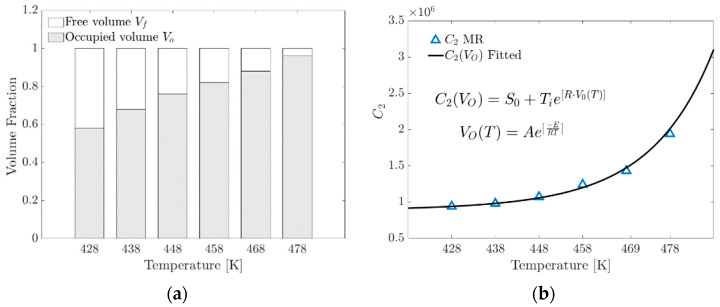
(**a**) Volume fraction distribution versus temperature. (**b**) Contrasting the $C_{2}$ parameter of the MR model with that calculated using the proposed exponential model.

Figure 11a graphically shows that as the temperature increases, the formation of crosslinks increases proportionally because the expansion of the linked chains within the unit cell tends to occupy the available volume $\left(FFV\right)$.

Figure 11b shows the parameter $C_{2}$ versus temperature. This plot shows the contrast of the parameter $C_{2}$ obtained from the fit with the MR model and the proposed model as a function of the occupied volume fraction.

Niu et al. [3] proposed an exponential-type model to determine the parameters of rubber material $C_{1}$ and $C_{2}$. They obtained good results with a mean deviation between 4.1% and 5.2% from the calculated results and combined with nonlinear autoregression (NAR). The degradation of crosslinking as a function of time is studied indirectly in the Niu et al. model; in the case of this investigation, the RM parameters are a function of temperature. The phenomenology is consistent because the empirical relationship between the two models focuses on studying elastomer crosslink evolution. With the model proposed in this research, a mean deviation of less than 3.5% was obtained. The values of the fitting parameters are shown in Table 2 (Equations (19), (21) and (22)).

Lengger et al. [11] correlated molecular-level crosslinking and chain entanglement as the main factors causing significant changes in the macroscopically observed mechanical properties of the polymer, i.e., increased stiffness. They also found that for parameters $C_{1}$ and $C_{2}$, there is a strong correlation with the reduction in the available volume, and these show exponential saturation when they reach approximately 88.6% of their maximum values.

In their research, Panyukov et al. [32] analyzed structures with highly overlapping and interconnected loops of a finite size, the conformation of combined chains in polymer networks and their influence on the resulting mechanical properties. In this investigation, in agreement with previous reports by Lengger and Panyukov and their respective collaborators, it is found that the parameters of the Mooney–Rivlin model depend strongly on the volume fraction of the polymer occupied by the crosslinking and entanglement of the chains. Table 3 shows the RM model parameters obtained from Equations (15), (20) and (22).

### 3.3. Crosslinking Density, Mechanical Stress and Their Relation

The temperature increase is proportional to the amount of energy required to achieve the deformation, according to the results of molecular dynamics simulations. This effect is manifested macroscopically as an increase in stiffness or the Young’s modulus (see Figure 7b). According to Saleesung et al. [36], Xie et al. [37], Papanikolaou et al. [2] and Bandyopadhyay et al. [38], with increasing crosslinking and decreasing free volume, the Young’s modulus and thus the tensile strength of the elastomer increases. Wang et al. [19] reported that the effect of crosslinking between the backbones or physical crosslinking increases the stiffness modulus and decreases the free volume fraction. Gomez et al. [4] found that at higher temperatures, the interactions of the molecules are weakened by the accelerated movement of the radius of gyration in the segments, causing the physical crosslinks to break at some point.

The mechanical properties of the polymer are related to moving molecular chains and varying free volumes. When mechanical stress is applied to EPDMs, the free volume distribution changes as the polymer chains reorganize due to stretching. This process continues until a steady state is reached. The increase in the crosslinking density is responsible for the expansion of the molecular chains within the unit cell, which implies a decrease in the free volume fraction, resulting in the increased stiffness and tensile strength of the EPDM elastomer.

**Figure 12:**
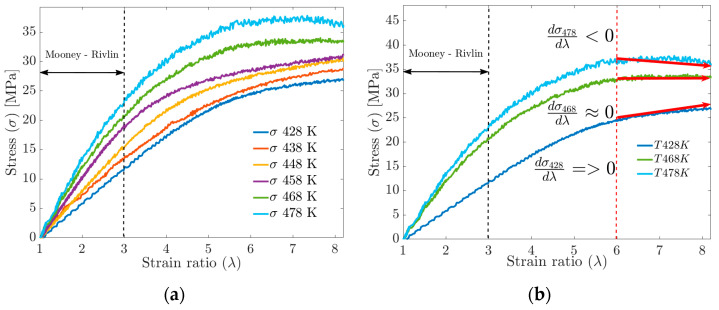
(**a**) Uniaxial tensile strength behavior vs. strain by MD. (**b**) Stiffness performance above 600% deformation.

For deformations greater than 300%, the MD simulation data no longer fit the Mooney–Rivlin model. By increasing the deformation up to 800%, significant changes in the S-s ratio can be observed in Figure 12a, such as the tensile strength, which increases with the increasing crosslink density but tends to stabilize at a given crosslink density. The mechanical strength of the cured rubber is also affected by the entanglement of the molecular chains. Rubbers with higher physical crosslinking (lower FFV) tend to have the highest tensile strength values, while materials with higher FFV have the lowest tensile strength values (see Figure 12a,b). Having said the above, Wang et al. [5] and Dijkhuis et al. [39] agree that free energy and the amount of physical crosslinking are related to changes in the stiffness of a material.

In plot 11b, for the S-s curves, in the strain ratio range above 600%, it is observed that at T=428 K, the tensile strength does not stabilize. At T=468 K, the tensile strength reaches a stable maximum value, while at T=478 K, the behavior can be interpreted as a fracture or failure of the material.

**Figure 13:**
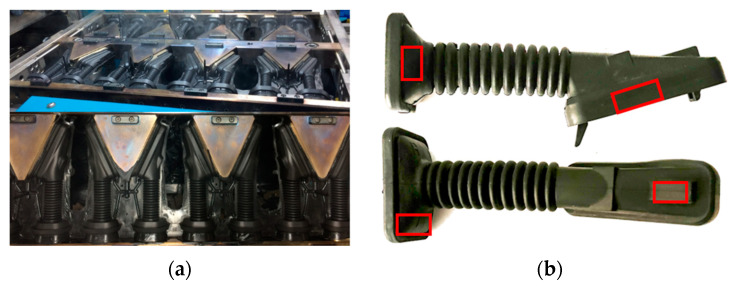
(**a**) Injection molding process in the door grommet; (**b**) Hardness testing measurement areas.

Physical tests have been carried out to verify the effect of the temperature on the hardness obtained during the injection molding process in the door grommet (see Figure 13a). Hardness tests were performed on the cured part by ASTM 1415-88 [40] using a PCE-DX-AS Rubber and Elastomer Hardness Tester (Shore A hardness 0–100+) and an International Rubber Hardness Degree Test (IRHD), with a measurement scale between 0 and 100, whose value is non-linear and related to the Young’s modulus (modulus of longitudinal elasticity) of the material. The International Hardness test is based on measurements of the penetration of a rigid ball into the rubber specimen under specified conditions. Figure 13b shows the areas of measurement.

The IRHD scale is defined as follows: if the Young’s modulus is zero, the IRHD hardness will be 0 and a material with an infinite Young’s modulus will correspond to an IRHD hardness of 100. Rubber hardness ranges from 30 to 90 IRHD, with 45 to 65 considered adequate for door grommet applications and 65 to 85 for bumper applications. Table 4 shows the hardness test results of the manufactured samples.

For soft materials, the hardness is between 10 and 35 IRHD and for tires, between 85 and 100 IRHD.

The results of the hardness tests (ASTM 1415-88 standard) on the Door Grommet component show that the hardness, and, therefore, the Young’s modulus, increases with the increasing temperature, which is consistent with the results obtained from the stress–strain ratio curves of the molecular dynamics simulation (see Figure 7a,b), where the change in the slope with the temperature can be seen, as well as with the data adjusted to the Mooney–Rivlin model (see Figure 9a). The results of the MR model can be correctly extrapolated to the Door Grommet applications since the deformation range does not exceed 200% in use.

The results of this research indicate that for elastomeric polymers, there is a temperature ($T_{u}$) at which a threshold crosslink density is reached. In an injection molding process below the threshold temperature, an under-cure phenomenon will occur, while in a process with a temperature above $T_{u}$, an over-cure phenomenon will occur.

If under-cure occurs, the consistency will be soft and may not return to its original dimensions when deformed over 600%. When over-cure occurs, it will crack when subjected to deformations above 600%. When curing occurs at temperatures near $T_{u}$ and the strain ratio exceeds 600%, the tensile strength reaches a stable maximum.

The mechanical behavior is reasonably consistent with the Mooney–Rivlin model for strain ratios of less than 300% in the temperature range tested.

## 4. Conclusions

The results presented in this research provide new molecular insights into the crosslinking mechanisms of elastomeric polymers and their influence on mechanical behavior, which could facilitate the design of rubbery products in applications such as door grommets. In combination with recent publications on tensile strength behavior using the Mooney–Rivlin model for rubbery polymers, they demonstrate the ability of MD simulations to elucidate complex phenomena associated with deformation and crosslinking behaviors in polymers.

The time-scale used in the molecular dynamics simulations might seem to be a limitation for the superposition of results; the phenomenological model presented in this research to predict the stress–strain behavior depends on the crosslink density, which can be understood macroscopically as vulcanization, so the time-scale is not an important issue. In this case, the molecular dynamics results allow us to perform multi-scale simulations.

This research provides evidence that the temperature during the curing process plays a preponderant role in the formation of crosslinks, resulting in a complex mixture of crosslinked structures (chemical and physical), as well as the movement of the backbone of the EPMD polymer trying to form clusters of crosslinks between the main chains, generating a significant conformational change, and a decrease in the free volume which translates into greater stiffness at higher temperatures.

The proposed model for determining Mooney–Rivlin parameters in elastomeric materials, which takes into account the effect of varying crosslink densities and volume fractions occupied by polymer chains as a function of temperature, provides a practical approach to analyze the impact on the mechanical performance of rubbery materials in applications such as door grommets and other types of seals. However, it is not limited to this, as any material that undergoes a curing process or/and large deformations can be analyzed with the techniques investigated here.

## Figures and Tables

**Table 1:** Crosslinking density, crosslinking rate and free volume fraction of EPDM compound at different temperatures.

*T* (*K*)	$N_{c}$ $\;10^{-3}(mol/{cm}^{3})$	${\dot{N}}_{c}$ $\;10^{-5}(mol/{cm}^{3}s)$	FFV
428	1.2302	0.1968	0.42
438	1.3815	0.3533	0.32
448	1.5052	0.6226	0.24
458	1.5949	1.0424	0.18
468	1.6591	1.8005	0.12
478	1.7215	3.4209	0.04

**Table 2:** Parameters of empirical models proposed for MR, Equations (19), (21) and (22).

$a_{0}$	$a_{1}$	$a_{2}$	$S_{0}$	A	$E\;(MPa\cdot {cm}^{3}/mol)$
−27.57 × 10^−3^	1.188 × 10^−4^	−1.204 × 10^−7^	0.8795 × 10^6^	54.4553	15.946 × 10^3^

**Table 3:** Parameters $C_{1}$ and $C_{2}$.

	Mooney–Rivlin		Proposed Models	
T (K)	$C_{1}$ $\;10^{6}(Pa)$	$C_{2}\;10^{6}(Pa)$	$C_{1}(T)\;10^{6}(Pa)$	$C_{2}\left(V_{O}\right)\;10^{6}(Pa)$
428	2.1854	0.9385	2.2130	0.9388
438	2.5105	0.9785	2.5465	0.9804
448	2.7921	1.0691	2.8233	1.0563
458	3.0346	1.2392	3.0014	1.1984
468	3.2421	1.4291	3.1382	1.4718
478	3.4186	1.9394	3.2998	2.0112

**Table 4:** ASTM 1415-88 hardness test.

	Hardness				
T	Test 1	Test 2	Test 3	Test 4	
(K)	Shore—A	Shore—A	Shore—A	Shore—A	IRHD
428	32.8	32.1	31.5	33.1	28
438	35.4	34.4	33.7	35.1	31
448	40.7	38.6	41.9	41.1	36
458	48.8	49.3	50.8	47.4	45
468	63.8	60.8	64.5	60.7	60
478	92.3	90.5	91.4	92.3	90

## Data Availability

The original contributions presented in the study are included in the article, further inquiries can be directed to the corresponding author.
